# Cross-cultural decoding of positive and negative non-linguistic emotion vocalizations

**DOI:** 10.3389/fpsyg.2013.00353

**Published:** 2013-07-30

**Authors:** Petri Laukka, Hillary Anger Elfenbein, Nela Söder, Henrik Nordström, Jean Althoff, Wanda Chui, Frederick K. Iraki, Thomas Rockstuhl, Nutankumar S. Thingujam

**Affiliations:** ^1^Department of Psychology, Stockholm UniversityStockholm, Sweden; ^2^Olin Business School, Washington University, St. LouisMO, USA; ^3^UQ Business School, University of QueenslandBrisbane, QLD, Australia; ^4^Haas School of Business, University of CaliforniaBerkeley, CA, USA; ^5^United States International UniversityNairobi, Kenya; ^6^Nanyang Business SchoolNanyang Technological University, Singapore; ^7^Department of Psychology, Sikkim UniversityGangtok, India

**Keywords:** affect bursts, cross-cultural, emotion recognition, non-verbal behavior, positive emotions, vocalizations

## Abstract

Which emotions are associated with universally recognized non-verbal signals?We address this issue by examining how reliably non-linguistic vocalizations (affect bursts) can convey emotions across cultures. Actors from India, Kenya, Singapore, and USA were instructed to produce vocalizations that would convey nine positive and nine negative emotions to listeners. The vocalizations were judged by Swedish listeners using a within-valence forced-choice procedure, where positive and negative emotions were judged in separate experiments. Results showed that listeners could recognize a wide range of positive and negative emotions with accuracy above chance. For positive emotions, we observed the highest recognition rates for relief, followed by lust, interest, serenity and positive surprise, with affection and pride receiving the lowest recognition rates. Anger, disgust, fear, sadness, and negative surprise received the highest recognition rates for negative emotions, with the lowest rates observed for guilt and shame. By way of summary, results showed that the voice can reveal both basic emotions and several positive emotions other than happiness across cultures, but self-conscious emotions such as guilt, pride, and shame seem not to be well recognized from non-linguistic vocalizations.

## Introduction

Studies of non-verbal emotion expression have provided crucial input to many of the central debates in emotion science. Controversies ranging from the universality of emotions (e.g., Ekman, 1993; Russell, 1994; Elfenbein, 2013), to the wider issue of how emotions should be conceptualized (e.g., Scherer, 1986; Ekman, 1992; Barrett, 2006), have all been fueled by data from studies of emotion expression. Here, we address a fundamental question raised in these debates—namely which emotions can be communicated across cultures—by examining how reliably the voice can convey cross-culturally a wide range of both positive and negative emotions.

The human voice is a rich source of emotional information and non-verbal vocal expressions come in two main forms, namely modifications of prosody (tone of voice) during speech (i.e., *prosodic expressions*), and through non-speech vocal sounds such as breathing sounds, crying, hums, grunts, laughter, shrieks, and sighs (i.e., *non-linguistic vocalizations*). Extensive reviews have established that prosodic expressions of basic emotions such as anger, fear, happiness, and sadness are conveyed by acoustic patterns of cues related to pitch, intensity, voice quality, and durations (Juslin and Laukka, 2003; Scherer, 2003). Several studies have further shown that decoders are able to infer the emotional content of prosodic expressions across languages and cultural boundaries with accuracy above chance (e.g., Kramer, 1964; Beier and Zautra, 1972; Albas et al., 1976; van Bezooijen et al., 1983; Graham et al., 2001; Scherer et al., 2001; Thompson and Balkwill, 2006; Bryant and Barrett, 2008; Pell et al., 2009). These studies suggest that perception of prosodic expressions has a universal component, although meta-analyses have also shown that communication is more accurate when judges rate expressions from their own culture compared with unfamiliar cultures (Elfenbein and Ambady, 2002; Juslin and Laukka, 2003).

Non-linguistic vocalizations [sometimes also referred to as affect bursts; see Scherer (1994)] differ from prosodic expressions in important ways. For example, speech requires highly precise and coordinated movement of the articulators (e.g., lips, tongue, and larynx) in order to transmit linguistic information, whereas non-linguistic vocalizations are not constrained by linguistic codes and thus do not require such precise articulations (Scott et al., 2009). This entails that non-linguistic vocalizations can exhibit larger ranges for many acoustic features than prosodic expressions—as evident by comparing, for example, pitch ranges in laughter vs. speech (Bachorowski et al., 2001). Compared to prosodic expressions, non-linguistic vocalizations may also be more strongly affected by physiological alterations (e.g., autonomic activation) to the appraisal of emotional situations and their effects on the vocal apparatus. Because vocal expressions are hypothesized to largely result from such emotion-related somatic alterations (see Scherer, 1986), non-linguistic vocalizations may be particularly suited for emotive communication.

However, compared to the large number of studies on prosodic expressions, relatively few studies have investigated emotion recognition from non-linguistic vocalizations (Schröder, 2003; Sauter and Scott, 2007; Belin et al., 2008; Hawk et al., 2009; Simon-Thomas et al., 2009; Sauter et al., 2010a; Lima et al., 2013). These studies show that decoders are generally accurate when judging basic emotions from non-linguistic vocalizations, often reaching higher recognition rates than for prosodic stimuli (e.g., Hawk et al., 2009). Some studies on vocalizations have also extended their coverage of emotions to include several emotions not generally viewed as basic. In particular, findings suggest that non-linguistic vocalizations may convey a wider palette of positive emotional states compared to facial expressions (Sauter and Scott, 2007; Simon-Thomas et al., 2009), as hypothesized by Ekman (1992). This suggests that different modalities of expression, such as facial and vocal expression, and perhaps also different varieties of expression within each modality, such as prosodic expressions and non-linguistic vocalizations, may be preferentially suited for expressing different emotions (see also Hawk et al., 2009; App et al., 2011).

It would seem that non-linguistic vocalizations, being unconstrained by conventions of language, would provide ideal stimuli for cross-cultural studies, but we are aware of very few previous studies on this topic. Sauter et al. (2010b) examined recognition of nine emotions, including basic emotions and additional positive emotions, across European English speaking individuals and individuals from remote, culturally isolated Namibian villages. They reported successful communication of basic emotions across cultural barriers, whereas recognition of positive emotions reached accuracy above chance mainly in within-group conditions. Koeda et al. (2013), in turn, let individuals from Canada and Japan rate Canadian vocalizations of basic emotions with regard to perceived levels of activation, valence and intensity, and reported some group differences in ratings of valence and intensity for both positive and negative emotions. Previous research has thus provided initial findings of both cultural similarities and differences, but further research is needed to establish the degree of cross-cultural variance and invariance of non-linguistic vocalizations.

In the present study we double the number of included emotions compared to previous studies and examine recognition of 18 emotions in cross-cultural conditions. By including the widest selection of emotions to date in a cross-cultural study, we aim to examine the limits of what non-linguistic vocalizations can reveal about emotion in a cross-cultural context. Notably, our selection of emotions includes equally many positive (affection, amusement, happiness, interest, sexual lust, peacefulness/serenity, pride, relief, and positive surprise) and negative (anger, contempt, disgust, distress, fear, guilt, sadness, shame, and negative surprise) emotions. Very few previous cross-cultural studies—regardless of expression modality—have examined recognition of positive emotional states beyond happiness, and our study will therefore provide novel clues about the universality of positive emotion expressions.

## Study 1—decoding of positive non-linguistic vocalizations

### Materials and methods

#### Vocal stimuli

We utilized non-linguistic vocalizations from the VENEC corpus, which is a large cross-cultural database of vocal emotion expressions portrayed by 100 professional actors (Laukka et al., 2010). The majority of stimuli in the VENEC corpus consist of prosodic expressions, but a subset of the actors also provided non-linguistic vocalizations, or affect bursts, and these stimuli are used in the present study. Actors from India, Kenya, Singapore, and USA were instructed to convey nine positive emotions (affection, amusement, happiness, interest, sexual lust, peacefulness/serenity, pride, relief, and positive surprise) by means of non-linguistic vocalizations. All vocalizations were intended to convey expressions with medium (moderately high) emotion intensity. Emotionally neutral vocalizations were also recorded, but these are not included in the current study.

The actors were instructed to express the emotions as convincingly as possible and in a similar way as in real emotional situations. To achieve this, the actors were first provided with scenarios describing typical situations in which each emotion may be elicited, based on current research on emotion appraisals (e.g., Ortony et al., 1988; Lazarus, 1991; Ellsworth and Scherer, 2003), and were then instructed to try to enact finding themselves in similar situations. As a further aid for producing convincing portrayals, they were also told to try to remember similar situations that they had experienced personally and that had evoked the specified emotions, and if possible to try to put themselves into the same emotional state of mind. Scherer and Bänziger (2010) have argued that a combination of scenarios and induction methods is likely to increase the authenticity and believability of the resulting portrayals because it discourages the use of stereotypical expressions.

The actors were free to choose whatever kind of human sounds that they thought fit for the purpose (e.g., breathing sounds, crying, hums, grunts, laughter, shrieks, and sighs). They were, however, told to avoid actual words (e.g., “heaven,” “no,” “yes”) and vocalizations with conventionalized semantic meaning (e.g., “yuck,” “ouch”), although non-linguistic interjections (e.g., “ah,” “er,” “hm,” “oh”) were allowed. Some actors nevertheless used words and these stimuli were excluded in an initial screening of the stimuli. Non-linguistic vocalizations were not recorded for each actor, and the number of emotions that each actor provided vocalizations for also varied. In total, our selection included 213 positive non-linguistic vocalizations from 41 actors (India, *N* = 9; Kenya, *N* = 11; Singapore, *N* = 7; and USA, *N* = 14), and contained approximately equally many portrayals of each emotion from each culture (see Table 1). The selection further included approximately the same number of stimuli by female and male actors in each condition.

**Table 1:** **Number of non-linguistic vocalizations for each emotion and culture**.

	**India**	**Kenya**	**Singapore**	**USA**	**Total**
**STUDY 1—POSITIVE EMOTIONS**					
Affection	6	5	6	6	23
Amusement	6	5	4	7	22
Happiness	7	6	5	7	25
Interest	6	6	6	7	25
Lust	6	6	5	7	24
Pride	6	4	6	7	23
Relief	6	6	5	7	24
Serenity	6	5	5	7	23
Surprise (positive)	6	5	6	7	24
Overall	55	48	48	62	213
**STUDY 2—NEGATIVE EMOTIONS**					
Anger	6	5	6	8	25
Contempt	6	6	5	8	25
Disgust	6	6	5	7	24
Distress	6	6	5	7	24
Fear	6	6	4	7	23
Guilt	6	6	4	7	23
Sadness	6	5	3	7	21
Shame	6	6	6	7	25
Surprise (negative)	6	5	6	7	24
Overall	54	51	44	65	214
Total	109	99	92	127	427

Recordings were conducted on location in each country (Pune, India; Nairobi, Kenya; Singapore, Singapore; and Berkeley, CA, USA), and the vocalizations were recorded directly onto a computer with 44 kHz sampling frequency using a high-quality microphone (sE Electronics USB2200A, Shanghai, China). The loudness of the stimuli varied widely—literally ranging from whispers to screams—and the amplitude of each stimulus was therefore peak normalized using *Adobe Audition* software (Adobe Systems Inc., San Jose, CA, USA). The normalization procedure controlled for differences in recording level between actors and softened the contrast between stimuli which would otherwise have been disturbingly loud or inaudibly quiet.

#### Participants and procedure

Twenty-nine Swedish individuals, mainly university students, took part in the study (20 women; mean age = 31 years). Participants judged the vocalizations of positive emotions by choosing one label which best represented the expression conveyed by each speech stimulus, and the alternatives they could choose from were the same as the nine intended expressions (affection, amusement, happiness, interest, lust, peacefulness, pride, relief, and positive surprise). All participants were provided dictionary definitions of each emotion, and also received the same emotion scenarios as did the actors, to make sure that they understood all of the included emotion labels.

Responses were scored as correct if the response matched the intended expressions of the emotion portrayals. Experiments were computerized and conducted individually using *MediaLab* software (Jarvis, 2008). Stimuli were presented in random order, and the participants were only allowed to listen to each stimulus once. The participants listened to stimuli through high-quality headphones, with the sound level kept constant across participants. Sessions lasted for ~40 min, and participants received course credits or a movie ticket voucher as compensation for their participation.

### Results

Table 2 shows the recognition rates and confusion patterns for positive emotions. The overall recognition rate was 39%, which is 3.5 times higher than the proportion expected by chance guessing (the chance level in a 9-alternative forced choice task is 11%; 1 out of 9). All emotions were recognized with accuracy above chance in at least some cultural conditions, as indicated by binomial tests. This suggests that a wide range of positive vocalizations were conveyed across cultures. Vocalizations of relief (mean recognition rate = 70%) were most accurately perceived, followed by lust (45%), interest (44%), serenity (43%), and positive surprise (42%). These emotions were not frequently confused with other states, although interest was sometimes confused with positive surprise, and serenity with relief.

**Table 2:** **Recognition rates and confusion patterns for non-linguistic vocalizations of nine positive emotions from four cultures**.

	**Intended emotion**									
**Judgment**	**Culture**	**Affection**	**Amusement**	**Happiness**	**Interest**	**Lust**	**Pride**	**Relief**	**Serenity**	**Surprise (positive)**
Affection	India	21^*^								
	Kenya	24^*^			20	11	14		13	
	Singapore	**18**							10	
	USA	**16**	12			10				
Amusement	India		**16**	27			10			10
	Kenya	21	45^*^	34						12
	Singapore		36^*^	30					10	11
	USA		34^*^	23						13
Happiness	India		18	35^*^						15
	Kenya	10	40	40^*^			18			25
	Singapore		23	29^*^						14
	USA		17	39^*^						19
Interest	India	21			51^*^		29			14
	Kenya	18			35^*^	10	18		10	
	Singapore	24			49^*^	12	39			
	USA				43^*^		25			
Lust	India	13				61^*^			19	
	Kenya					26^*^		13		
	Singapore	16				41^*^			13	10
	USA	19				48^*^		10	16	12
Pride	India	10		15			19^*^			
	Kenya						26^*^			
	Singapore			13			**10**			
	USA		16	10			33^*^			
Relief	India							67^*^	21	
	Kenya					13		68^*^	26	
	Singapore			14		14	20	75^*^		
	USA	24						69^*^	30	14
Serenity	India					18		19	48^*^	
	Kenya	11							25^*^	
	Singapore	17				14		12	49^*^	
	USA	30			7	24		14	46^*^	
Surprise (positive)	India		43	13	29		13			44^*^
	Kenya			10	20	10				46^*^
	Singapore		17		19		14			47^*^
	USA			12	31		18			33^*^

Note: The recognition rates (percentage accuracy) for which the expression portrayed is the same as the expression judged are shown in the diagonal cells (marked in bold typeface). Asterisks denote recognition rates higher than what would be expected by chance guessing (11%), as indicated by binomial tests (ps < 0.05, Bonferroni corrected; ps < 0.001, uncorrected). Blank cells indicate misclassification rates of less than 10%.

Happiness (36%) and amusement (32%) were symmetrically confused with each other at a level equal to accurate decoding proportion, which suggests that vocalizations of these states are not easy to separate. Given the conceptual similarity between these states this was hardly a surprising finding, and a combined happiness/amusement category received 60% accuracy. At the bottom end of recognizability, we found pride (22%) and affection (20%). Although recognized with above-chance accuracy in some conditions, these emotions were frequently misclassified, and vocalizations of both pride and affection were most commonly confused with interest.

Inspection of the recognition rates as a function of speaker culture further revealed that both recognition and confusion patterns were similar across all four cultures (see Table 2). This suggests cross-cultural consistency with regard to which emotions were easy or hard to recognize, and which emotions were confused with each other and which were not. Nevertheless, some emotions were only recognized in some, but not in other, cultural conditions. For example, Swedish listeners did not accurately perceive amusement vocalizations from Indian stimuli, but instead judged them as surprised sounding. However, it is difficult to interpret such group differences, because they may result from group effects not having to do with culture *per se* (e.g., the Indian actors may simply not have been as successful in portraying amusement compared to actors from other cultures).

## Study 2—decoding of negative non-linguistic vocalizations

### Materials and methods

#### Vocal stimuli

Non-linguistic vocalizations of nine negative emotions (anger, contempt, disgust, distress/pain, fear, guilt, sadness, shame, and negative surprise) portrayed by professional actors from India, Kenya, Singapore, and USA served as stimuli in Study 2. The vocalizations were selected from the VENEC corpus (Laukka et al., 2010) and were collected using the same methods as described for Study 1. In total, the selection contained 214 negative emotional vocalizations from 40 actors (India, *N* = 8; Kenya, *N* = 10; Singapore, *N* = 7; and USA, *N* = 15), see Table 1 for details.

#### Participants and procedure

We used the same judgment procedures in Study 2, as previously described for Study 1, except that we presented negative vocalizations and response alternatives. Twenty-eight Swedish individuals (18 women; mean age = 31 years) judged the expressed emotion of each presented stimulus, by choosing one from nine alternatives (anger, contempt, disgust, distress, fear, guilt, sadness, shame, and negative surprise). Four of the participants had previously taken part in Study 1.

### Results

Recognition rates and confusion patterns for negative emotions are presented in Table 3. For negative emotions, the overall recognition rate was approximately four times higher than chance at 45%. Similar to Study 1, we conducted binomial tests to test whether the proportion of participants who chose the correct response alternative for each emotion was higher than the proportion that would be expected by chance guessing. All emotions were recognized with accuracy above chance in at least some conditions—which suggests that a wide range of negative emotions can be expressed cross-culturally through the voice. Disgust (mean recognition rate = 63%) was the best recognized emotion, followed by anger (57%), fear (57%), sadness (56%), negative surprise (53%), and contempt (44%). These emotions were seldom confused with other states, although contempt was sometimes confused with negative surprise.

**Table 3:** **Recognition rates and confusion patterns for non-linguistic vocalizations of nine negative emotions from four cultures**.

	**Intended emotion**									
**Judgment**	**Culture**	**Anger**	**Contempt**	**Disgust**	**Distress**	**Fear**	**Guilt**	**Sadness**	**Shame**	**Surprise (negative)**
Anger	India	42^*^	14							
	Kenya	38^*^	10	12						13
	Singapore	46^*^								
	USA	88^*^								
Contempt	India	20	52^*^				18		16	
	Kenya	30	29^*^	12					12	11
	Singapore		57^*^		20					
	USA		42^*^							
Disgust	India			40^*^					12	
	Kenya		15	58^*^					11	
	Singapore			81^*^	12					
	USA			67^*^						13
Distress	India			19	52^*^	12		11		
	Kenya		12	12	52^*^	12	26	16	17	22
	Singapore	16			**15**	25	10	15	24	
	USA			12	34^*^	19	23		29	16
Fear	India					60^*^		11		
	Kenya					60^*^		16		11
	Singapore				24	55^*^		15	14	
	USA				27	58^*^		15		
Guilt	India						19^*^		18	
	Kenya						**14**		17	
	Singapore						29^*^	14	15	
	USA						23^*^		15	
Sadness	India				21			65^*^		
	Kenya				21			58^*^		10
	Singapore							27^*^		
	USA				14	12		61^*^		
Shame	India						17		22^*^	
	Kenya						22		21^*^	
	Singapore						28	13	**16**	
	USA						26		24^*^	
Surprise (negative)	India	11	18	12		11	30		20	80^*^
	Kenya		18			11	18		11	**18**
	Singapore	18	26				21		17	68^*^
	USA		25				12		13	42^*^

Note. The recognition rates (percentage accuracy) for which the expression portrayed is the same as the expression judged are shown in the diagonal cells (marked in bold typeface). Asterisks denote recognition rates higher than what would be expected by chance guessing (11%), as indicated by binomial tests (ps < 0.05, Bonferroni corrected; ps < 0.001, uncorrected). Blank cells indicate misclassification rates of less than 10%.

Distress (33%) was frequently confused with both fear and sadness, which suggests that distress vocalizations may show some overlap with these emotions. The most frequently observed confusions occurred symmetrically between shame (mean recognition rate = 21%) and guilt (mean recognition rate = 20%). A joint shame/guilt category indeed received 40% accuracy, which could be interpreted as evidence for the notion that the voice can reveal a negative self-conscious emotion category. However, both shame and guilt were frequently confused also with other emotions, such as distress and negative surprise, which instead indicates that they may not be associated with distinct vocal signals.

Table 3 further displays recognition rates as a function of speaker culture, and inspection revealed substantial cross-cultural consistency with regard to both recognition and confusion patterns. However, some cultural variability could also be observed. For example, Swedish listeners frequently confused distress vocalizations from India and Kenya with sadness, whereas distress vocalizations from Singapore and USA were instead confused with fear. However, as previously explained, we cannot know if group differences are caused by cultural factors or factors unrelated to culture.

## Discussion

The present results establish non-linguistic vocalizations as a rich and nuanced source of emotional signals. Across two studies, our results suggest that the voice can convey a wide range of positive (Study 1) as well as negative (Study 2) emotions across cultures. More specifically, we observed above-chance cross-cultural recognition of basic emotions such as anger, contempt, disgust, fear, happiness, sadness, and surprise. Notably, we also observed for the first time above-chance recognition of several positive emotions other than happiness—such as interest, lust, relief, and serenity—in a cross-cultural context. However, not all emotions were equally recognizable across cultures and we observed only modest recognition rates for affection, guilt, pride, and shame. The implications of these findings are discussed below in relation to the larger issue about which emotions are associated with universally recognized expressions.

Findings of universality in emotion expression are traditionally interpreted as support for the proposition that emotion expressions are based on biologically driven evolved mechanisms (e.g., Ekman, 1992), although this view also has its critics (e.g., Barrett, 2006). Non-linguistic vocalizations are often considered an especially “primitive” form of human emotion signaling that is functional already at the time of birth and that in many ways resembles animal expressions more than human speech (Owren et al., 2010; Briefer, 2012). Thus, it may be hypothesized that cross-culturally communicable vocalizations may, at least to a certain extent, be based on evolved biologically driven mechanisms (e.g., Ekman, 1992), such as physiological effects of emotion appraisals on the voice production apparatus (Scherer, 1986). Our observation of above-chance recognition of basic emotions corroborates findings from the sole previous cross-cultural study on non-linguistic vocalizations by Sauter et al. (2010b), as well as previous studies on prosodic and facial expressions (e.g., Elfenbein and Ambady, 2002; Juslin and Laukka, 2003), and suggests that basic emotion vocalizations have a universal component.

We included a wide selection of positive emotions, and our observation of above-chance recognition of positive states other than happiness expands upon previous studies conducted in a within-cultural context (e.g., Simon-Thomas et al., 2009). The finding of a universal component to positive emotion vocalizations may appear contrary to the previous findings of Sauter et al. (2010b), who reported largely non-significant cross-cultural recognition for positive emotions. However, the distinctions between different positive emotions are not well understood, and as a consequence different studies have included different positive states. Between our study and Sauter et al. (2010b), the only common positive emotions were amusement and relief. Whereas Sauter et al. (2010b) observed cross-cultural recognition for amusement (which they viewed as a basic emotion) but not relief, we instead observed above-chance recognition for both emotions (although amusement was frequently confused with happiness). The main difference between studies thus concerns recognition of relief only, and may have been caused by idiosyncratic differences in the sets of expressive stimuli used in respective study. Despite the fact that expressors and perceivers in our study came from different continents, it also remains a possibility that the cultural distances may have been larger in Sauter et al. (2010b) compared to our study.

Similar to our observations, previous within-cultural studies have also reported modest recognition rates for affection, guilt, pride, and shame (Hawk et al., 2009; Simon-Thomas et al., 2009). Taken together, current evidence thus suggests that these emotions may not be associated with highly distinct vocalizations. Guilt, pride, and shame involve reflection upon and evaluation of the self (Tangney and Tracy, 2012), which makes these emotions more dependent on complex cognitive skills compared to basic emotions. Cultures vary regarding how the self is conceptualized (Markus and Kitayama, 1991), and this may lead to culture-specific interpretations of situations particularly relevant for self-conscious emotions such pride and shame (Imada and Ellsworth, 2011). There is thus a possibility that cultural variance may be especially salient for expressions of self-conscious emotions. Although we cannot draw this conclusion based on our current data—because we did not assess emotion recognition in both within- and cross-cultural conditions—this remains an interesting question for future studies. However, evidence also suggests that pride and shame are expressed in a similar fashion cross-culturally through facial and bodily cues (Tracy and Matsumoto, 2008), which leaves open the possibility that they may have distinct expressions through other modalities than the voice.

Comparing our results to previous studies on prosodic expressions, we note that disgust vocalizations received high accuracy rates in our study (as well as in most previous vocalization studies; e.g., Schröder, 2003), whereas disgust is often poorly recognized from prosodic stimuli (e.g., Banse and Scherer, 1996). This suggests that some emotions may be better decoded from vocalizations versus emotional prosody, and future studies could perform direct comparisons to establish which emotions are preferentially recognized from which type of expression. Hawk et al. (2009) reported higher accuracy for vocalizations compared to prosodic expressions for a range of mainly negative emotions, but comparisons for positive emotions are currently missing. Such studies could also include other expression channels—such as facial, bodily, olfactory, and tactile cues—in order to provide a foundation for understanding of which emotions are preferentially expressed through which modalities (e.g., App et al., 2011).

Our investigation also has several limitations which merit consideration. Recent cross-cultural studies on decoding of facial (Elfenbein et al., 2007), musical (Laukka et al., 2013), and vocal (Sauter et al., 2010b) expressions have reported evidence for an in-group advantage to the effect that decoders perform better for expressions from a familiar versus an unfamiliar culture. However, we only assessed decoding in cross-cultural conditions, which precluded investigation of an in-group advantage in the current study. The lack of a within-cultural baseline rate, together with the small number of stimuli in each emotion × culture cell, also prevents a meaningful comparison of recognition rates between cultures—because differences may have been caused by group effects other than culture. We further assessed positive and negative emotions in separate forced-choice experiments in order to avoid fatigue in the participants and to keep the number of response options at a manageable level. However, this design prevented us from investigating possible confusions between positive and negative expressions. The use of a forced-choice format has also been criticized on the grounds that it may lead to inflated recognition rates by enabling judges to use informed guessing strategies to a certain extent (e.g., Russell, 1994). Finally, we used portrayed rather than spontaneous vocalizations, whereas some previous studies have reported that acted expressions may be more prototypical and intense than spontaneous expressions (e.g., Laukka et al., 2012). We are addressing the question of a possible in-group advantage in ongoing cross-cultural judgment studies, and would welcome future studies that consider effects of the format of the judgment task and type of expressive stimuli on cross-cultural emotion decoding (e.g., Jürgens et al., 2013).

Non-linguistic vocalizations are heterogeneous and contain many different types of human sounds, and our sample can only represent a limited subset of all possible vocalizations. We instructed the actors to avoid the use of vocalizations with conventionalized semantic meaning, because the production and recognition of emblematic affect expressions is hypothesized to be strongly culture-dependent (see Scherer, 1994). However, it remains a possibility that some of our vocal stimuli nevertheless contained such culture-dependent information, and this may have reduced recognition accuracy for some emotion × culture combinations. Our study was limited to decoding, but future studies could also investigate how different emotions are encoded in the acoustic properties (such as pitch, intensity, voice quality, and durations; Sauter et al., 2010a; Lima et al., 2013) and in the segmental-phonemic structure (Schröder, 2003) of non-linguistic vocalizations. Currently, cross-cultural studies linking encoding and decoding are missing, but such studies have the potential to reveal which aspects of non-linguistic emotion vocalizations are culturally invariant and which rely on culture-dependent templates.

To conclude, our results show that non-linguistic vocalizations can convey detailed emotional information—not limited to the usual basic emotions, or activation and valence dimensions—to listeners across cultures. We therefore propose that vocalizations may provide ideal stimuli for theory development and applied research in emotion science. Compared to negative emotions, positive emotions have received much less attention, and as a result knowledge about the cognitive appraisals underlying different positive states, and their effects on physiology, is limited. Because vocalizations seem to convey a particularly wide range of positive states, we suggest that studies on non-linguistic vocalizations provide a promising avenue for investigating the distinctiveness of positive emotions.

### Conflict of interest statement

The authors declare that the research was conducted in the absence of any commercial or financial relationships that could be construed as a potential conflict of interest.
